# Autophagy Induced *FHL2* Upregulation Promotes IL-6 Production by Activating the NF-κB Pathway in Mouse Aortic Endothelial Cells after Exposure to PM2.5

**DOI:** 10.3390/ijms18071484

**Published:** 2017-07-17

**Authors:** Wen-Rong Xia, Wenliang Fu, Qin Wang, Xiaoming Zhu, Wei-Wei Xing, Min Wang, Dong-Qun Xu, Dong-Gang Xu

**Affiliations:** 1 Laboratory of Genomic Engineering, Beijing Institute of Basic Medical Sciences, Beijing 100000, China; xiawenrong@sina.com (W.-R.X.); fwl86@139.com (W.F.); kitty66cat@126.com (X.Z.); huozinangua@163.com (W.-W.X.); wmmm12@sina.cn (M.W.); 2 Institute for Environmental Health and Related Product Safety, Chinese center for disease control, Beijing 100000, China; wangqinbj@163.com

**Keywords:** PM2.5, *FHL2*, IL-6, NF-κB, autophagy, mouse aortic endothelial cells

## Abstract

Epidemiological and clinical studies have increasingly shown that fine particulate matter (PM2.5) is associated with cardiovascular morbidity and mortality, which share the common feature of PM2.5-induced vascular inflammation; however, the underlying mechanisms of how PM2.5 triggers increased inflammatory response in vascular endothelial cells are not well understood. After treating mouse aortic endothelial cells (MAECs) with different concentrations of PM2.5, we assessed interleukin (IL)-6 and four and a half LIM domains 2 (*FHL2*) expression in cell supernatant by enzyme-linked immunosorbent assay and Western blot, respectively, as well as activation of nuclear factor (NF)-κB and immune-response signaling pathways. Additionally, changes in pathway activation, IL-6 expression, and autophagy were evaluated under PM2.5 exposure, following *FHL2* knockdown with small interfering RNA. Our results indicated that PM2.5 exposure induced *FHL2* expression and IL-6 secretion, as well as activation of pathways associated with immune response. Additionally, following *FHL2* knockdown, the activation of NF-κB-related pathways and IL-6 secretion was inhibited under PM2.5 exposure, although the Akt- and p38-signaling pathways were not affected. Furthermore, PM2.5 exposure induced autophagy, whereas autophagy inhibition eventually inhibited PM2.5-induced *FHL2* expression. These findings suggested a novel link between autophagy induced *FHL2* upregulation and IL-6 production in MAECs under PM2.5 exposure.

## 1. Introduction

Epidemiological and clinical studies increasingly show that exposure to particulate matter with an aerodynamic diameter ≤ 2.5 µm (PM2.5) constitutes a risk factor associated with cardiovascular morbidity and mortality [1]. Cohort studies also found that risk of cardiovascular events is associated with increased blood levels of inflammatory cytokines, and that PM2.5 is a promoter of systemic inflammation and increased circulating levels of inflammatory cytokines [2,3,4]. After inhalation and deposition in the epithelium of the respiratory tract and lungs, particles are capable of moving into interstitial spaces between cells, followed by induction of a variety of pro-inflammatory cytokines, including monocyte chemoattractant protein 1, macrophage inflammatory protein 1α/β, interleukin (IL)-6 and IL-1β, and markers of endothelial adhesion, such as soluble intercellular adhesion molecule 1 (ICAM-1) and soluble vascular cellular adhesion molecule 1 (VCAM-1), that can lead to endothelial dysfunction [5,6]. Endothelial cells that form new blood vessels play an important role in the pathophysiology of cardiovascular diseases [7]. Therefore, understanding how PM2.5 triggers inflammatory responses associated with vascular endothelial dysfunction is crucial for the study of PM2.5 toxicity; however, the mechanisms underlying associations between PM2.5 and increased vascular inflammatory response have not been clearly defined.

Four and a half LIM domains 2 (*FHL2*) is a member of the FHL family of proteins, and is involved in interactions with various proteins and regulation of multiple transcription factors (TFs), cytoskeletal proteins, and enzymes. *FHL2* plays an important role in regulating inflammation, angiogenesis, and the cardiovascular system. Following stimulation with tumor necrosis factor-α, *FHL2* downregulation in skeletal muscle cells inhibits the IL-6 secretion [8]. Additionally, *FHL2* promotes activation of nuclear factor (NF)-κB in gastric cancer, liver regeneration, and liver cancer [9,10], and upregulated *FHL2* expression in human liver samples is significantly associated with increased inflammatory response and liver cirrhosis [11]. Moreover, FHL1–3 participate in the phosphorylation of mothers against decapentaplegic homolog-2, which activates the inflammatory response through the mitogen-activated protein kinase signaling pathway [12].

In both myeloid and vascular cells, *FHL2* also plays an important role in atherosclerosis by promoting proinflammatory chemokine production, adhesion-molecule expression, and proinflammatory monocyte recruitment [13], and *FHL2* deletion attenuates the formation of atherosclerotic lesions induced by a cholesterol-enriched diet [14]. Furthermore, *FHL2* was originally identified in the heart, and the association of *FHL2* mutations with human cardiac hypertrophy reinforce both its importance in the heart and its relevance to human cardiac disease [15,16,17]. Recent studies have focused on the role of *FHL2 in blood vessel walls, where it is involved in vascular lesion formation, and development of atherosclerosis [18].*

These reports suggest that *FHL2* might play an important role in the process of vascular cell dysfunction and cardiovascular disease by regulating the inflammatory response. In this study, we investigated the expression of *FHL2* and IL-6 following exposure to different concentrations of PM2.5, to determine the role of *FHL2 in promoting IL-6 expression via inflammatory* signaling pathways. Furthermore, particulate matter exposure induces the autophagy of macrophages, lung cancer cells, BEAS-2B cells, and human lung epithelial A549 cells [19,20,21,22]; however, the ability of PM2.5 to induce autophagy, and the relationship between *FHL2* and autophagy in mouse aortic endothelial cells (MAECs), remains unknown. Our results showed that PM2.5 induced autophagy in MAECs and might be involved in the *FHL2*-activated NF-κB pathway, and IL-6 upregulation.

## 2. Results

### 2.1. Characteristics of PM2.5 Components and Mass Concentration in Winter from a Beijing Urban Area

Table 1 shows the concentration range of PM2.5 and its adsorbed metal elements, collected from October to December 2015 in Beijing city.

### 2.2. PM2.5 Exposure Promotes FHL2 Expression and Activates Signaling Pathways in MAECs

**Figure 1:**
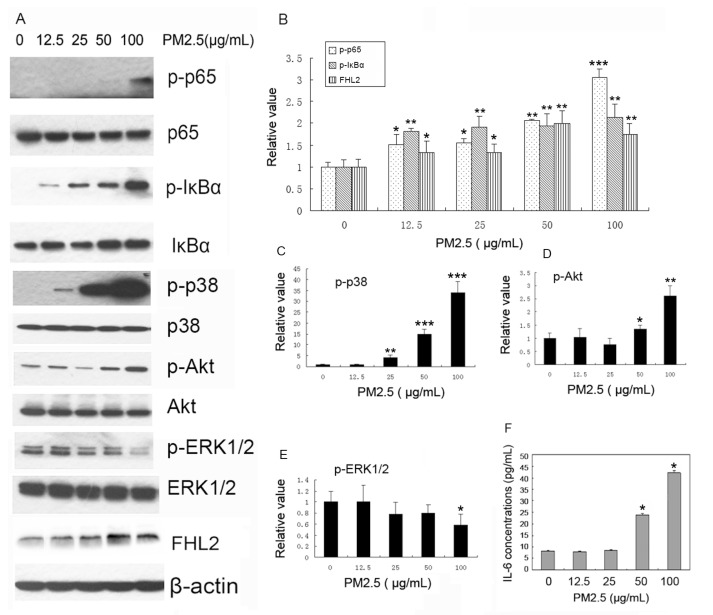
Activation of NF-κB and *FHL2* upregulation by PM2.5. (**A**) Cells were incubated with 0, 12.5, 25, 50, and 100 µg/mL PM2.5 for 24 h, and phosphorylated p65 (p-p65), p65, phosphorylated IκBα (p-IκBα), IκBα, phosphorylated p38(p-p38), p38, phosphorylated Akt (p-Akt), Akt, phosphorylated ERK1/2 (p-ERK1/2), ERK1/2, *FHL2*, and β-actin were detected in PM2.5-treated mouse aortic endothelial cells (MAECs). (*n* = 3); (**B**–**E**) Band quantifications for western blot analysis. * *p* < 0.05; ** *p* < 0.01; *** *p* < 0.001; (**F**) IL-6 expression detected in MEAC supernatant treated with different concentrations of PM2.5 according to ELISA.

To investigate the mechanism of PM2.5-induced vascular injury mediated by *FHL2*, MAECs were stimulated with different concentrations of PM2.5. We observed that *FHL2* expression was upregulated, along with increases in PM2.5 concentration, and that PM2.5 also promoted p65 and IκBα phosphorylation in a concentration-dependent manner. Additionally, PM2.5 exposure promoted p38 and Akt phosphorylation, but significantly inhibited extracellular signaling kinase (ERK)1/2 phosphorylation at concentrations of 100 µg/mL (Figure 1A–E). Furthermore, ELISA results showed that exposure to both 50 and 100 µg/mL PM2.5 significantly upregulated IL-6 expression in MEACs relative to controls (*p* < 0.01) (Figure 1F). These results demonstrated that *FHL2* might facilitate PM2.5-induced vascular injury by activating NF-κB and the p38- and Akt-signaling pathways, and promoting the production of the inflammatory cytokines.

### 2.3. FHL2 Knockdown Inhibits PM2.5-Activated NF-κB Signaling

**Figure 2:**
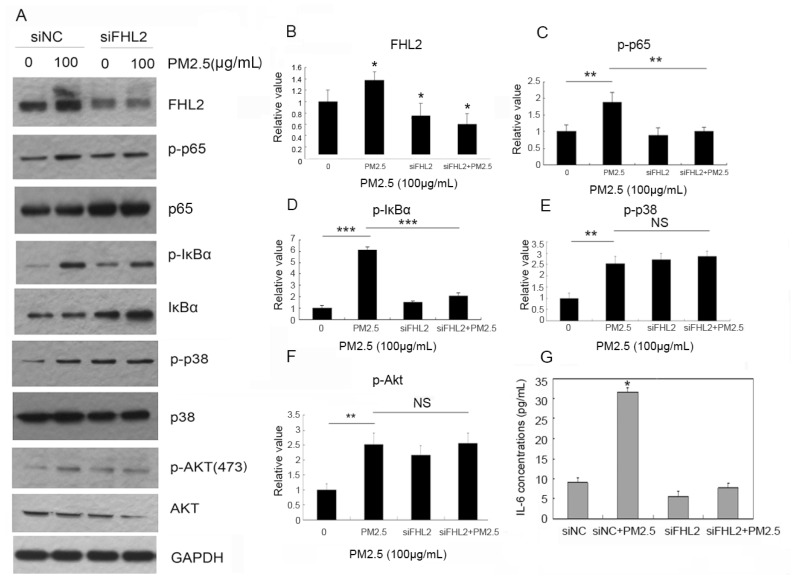
*FHL2* mediates PM2.5-induced NF-κB activation. (**A**) Cells were transfected with si*FHL2* or negative control siRNA (siNC) for 48 h, followed by exposure to PM2.5 at a final concentration of 100 µg/mL for another 24 h. *FHL2* protein levels, as well as those of phosphorylated p65, p65, phosphorylated IκBα, IκBα, and β-actin, were detected in whole-cell lysates (*n* = 3); (**B**–**F**) Band quantifications for western blot analysis; (**G**) IL-6 expression detected by ELISA. * *p* < 0.05; ** *p* < 0.01; *** *p* < 0.001. NS, no significance.

To determine *FHL2* involvement in PM2.5-activated signaling, *FHL2* expression was knocked down in MEACs by transfection of siRNA specific for *FHL2* (si*FHL2*). After *FHL2* knockdown, cells were stimulated with PM2.5 (100 µg/mL) for 24 h, and proteins were extracted from the cellular supernatant fraction for analysis. Our results showed that PM2.5 stimulation alone was sufficient to upregulate p65 and IκBα phosphorylation, whereas si*FHL2* transfection effectively knocked down *FHL2* expression and inhibited PM2.5-induced p65 and IκBα phosphorylation. However, PM2.5 exposure did not activate the p38- and Akt-signaling pathways in an *FHL2*-dependent manner (Figure 2A–F). These results suggested that *FHL2* mediated PM2.5-activated NF-κB signaling. Furthermore, ELISA detection of IL-6 expression showed that PM2.5 (100 µg/mL) exposure upregulated IL-6 expression, whereas attenuated *FHL2* expression inhibited PM2.5-induced IL-6 secretion (Figure 2G. These data suggested that PM2.5 exposure activated the NF-κB-signaling pathway by upregulating *FHL2* expression of *FHL2*, thereby inducing IL-6 secretion.

### 2.4. PM2.5 Exposure Induces Autophagy in MAECs

**Figure 3:**
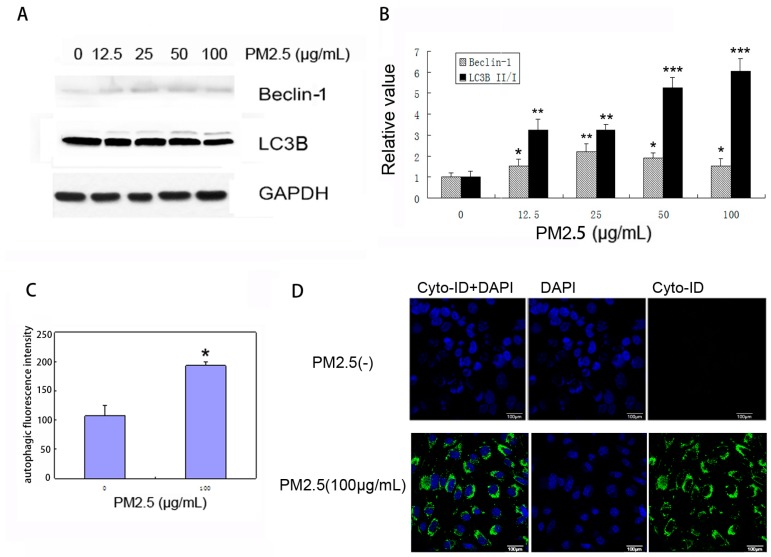
PM2.5 exposure induces autophagy in MAECs. (**A**) Cells were exposed to 0, 12.5, 25, 50, or 100 μg/mL PM2.5 for 24 h, and the expression of LC3B, Beclin-1, and GAPDH were examined (*n* = 3); (**B**) Bands quantified for Western blot analysis. * *p* < 0.05; ** *p* < 0.01; *** *p* < 0.001; (**C**) MAECs were treated with PM2.5 (100 µg/mL) for 24 h and stained with Cyto-ID reagent, followed by collection and flow cytometric analysis to quantitatively measure autophagic fluorescence intensity (*n* = 3). * *p* < 0.05. (**D**) MAECs were exposed to PM2.5 as described in (**C**), and autophagy was examined by confocal microscopy after cell staining. Scale bar = 100 µm.

As shown in Figure 3A–B, we observed dose-dependent upregulation of the LC3B-II:LC3B-I ratio and Beclin-1 expression in MAECs exposed to PM2.5, suggesting enhanced autophagosome synthesis in response to PM2.5 stimulation. We then measured autophagic activity in PM2.5-treated MAECs and analyzed cell population by flow cytometry (Figure 3C). Upon staining MAECs with Cyto-ID Green, we observed a significant induction in autophagic activity inside cells at 24 h after PM2.5 exposure, based on a green-fluorescent signal that accumulated in spherical vacuoles at the perinuclear region of PM2.5-treated cells (Figure 3D). By contrast, no obvious signal was detected in untreated MAECs under the same conditions.

### 2.5. Inhibition of Autophagy Downregulates PM2.5-Induced FHL2 Expression

**Figure 4:**
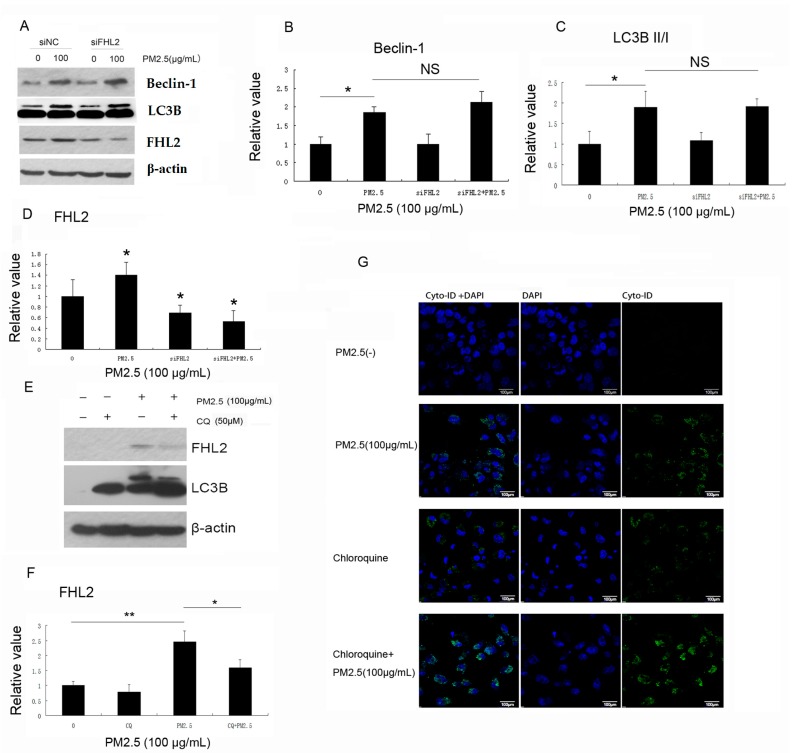
The relationship between autophagy and *FHL2* expression. (**A**) MAECs were transfected with siFHL3 or siNC for 48 h and exposed to 100 µg/mL PM2.5 for another 24 h, and protein levels of *FHL2*, LC3B, and Beclin-1 were detected in whole-cell lysates (*n* = 3); (**B**–**D**,**F**) Bands quantifying Western blot results; (**E**) MAECs were pretreated with CQ for 1 h, followed by exposure to PM2.5 (100 µg/mL) and analysis of *FHL2* expression 24 h after exposure, (*n* = 3). * *p* < 0.05; ** *p* < 0.01; (**G**) MAECs were treated with CQ or PM2.5 as described in (**E**), and autophagy was examined by confocal microscopy after staining with Cyto-ID Green. DAPI (4′,6-diamidino-2-phenylindole) is commonly used for nuclear staining. Scale bar = 100 µm. NS, no significance.

Because PM2.5 induces autophagy while upregulating *FHL2* expression, with both activities regulating NF-κB signaling, we investigated potential regulatory functions between *FHL2* and autophagy. Our results showed that PM2.5 exposure alone upregulated Beclin-1 expression and increased the LC3BII:LCB3-I ratio, and that these were unaffected by *FHL2* knockdown. These data suggested that PM2.5 did not induce autophagy via *FHL2* upregulation (Figure 4A–D).

Chloroquine (CQ) is a small molecule modulator that passively diffuses into the lysosome, and becomes trapped upon protonation. CQ administration also causes an increase in lysosomal pH, which inhibits lysosome function and blocks fusion of the autophagosome with the lysosome. Here, CQ was incubated with MEACs for 1 h prior to PM2.5 exposure. After incubation for 24 h, cell growth was assessed via immunoblot and immunofluorescence. Figure 4E shows that PM2.5 exposure alone was sufficient to upregulate LC3B and *FHL2* expression; however, CQ-mediated inhibition of autophagy inhibited PM2.5-induced *FHL2* expression, which was verified by immunofluorescence staining, revealing substantial increases in green fluorescence following exposure to PM2.5 accompanied by CQ treatment (Figure 4G). These results suggested that PM2.5 exposure upregulated *FHL2* expression through induction of autophagy.

## 3. Discussion

Epidemiological studies have reported associations between particulate matter and cardiovascular issues. PM2.5 exposure induces cardiovascular injury by increased endothelial cell apoptosis and systemic inflammation [5]. IL-6, an important inflammatory factor, is related to higher risk of major bleeding and death associated with cardiovascular events [23], and constitutes a regulator of vascular inflammation during cardiac transplantation [24]. Additionally, IL-6 release represents a key event in the onset and progression of endothelial dysfunction in diabetic vascular complications [25]. Therefore, upregulation of IL-6 expression is an important factor associated with vascular injury, with PM2.5 exposure capable of promoting IL-6 expression. However, the mechanisms of how to regulate PM2.5-induced IL-6 expression remain unknown.

PM2.5 regulates inflammatory responses by activating multiple signaling pathways. Inflammatory injury in rat lungs exposed to PM2.5 and SO_2_ is associated with p38/NF-κB-signaling pathway activation [26], and PM2.5-induced oxidative stress increases ICAM-1 and VCAM-1 expression in human endothelial cells via an ERK/Akt/NF-κB-dependent pathway [27]. PM2.5 exposure also results in a significant increase in the expression of inflammatory genes and activates p38- and ERK-signaling pathways in the liver of the KKAy mice [28]. Moreover, recent studies showed that signaling related to Akt, ERK, NF-κB, and p38 is activated, and results in PM2.5-induced alterations in inflammatory responses. In our study, we found that PM2.5 exposure activated NF-κB, p38, and Akt signaling while promoting *FHL2* expression and IL-6 secretion. *FHL2* is an important factor involved in regulating inflammatory factors; however, we observed that inhibition of *FHL2* expression only suppressed PM2.5-activated NF-κB signaling and IL-6 secretion, but did not affect p38 or Akt signaling. These results indicated that *FHL2*/NF-κB plays an important regulatory role in elevated IL-6 secretion induced by exposure to PM2.5.

Interestingly, we found that PM2.5 exposure induced autophagy in MAECs. Autophagy is a conserved, multi-step process associated with lysosomal degradation of proteins, organelles, and other macromolecules, and induced in response to a wide range of stressful conditions (physical, chemical, or metabolic) to maintain cellular homeostasis [29]. Its pivotal role in maintaining inflammatory homeostasis relates to dysfunctions in the autophagic process, with important pathological consequences. [30]. PM2.5 exposure induces autophagy, which contributes to endothelial apoptosis and dysfunction. [21,31]. Autophagy is required for hepatitis B virus X protein-induced NF-κB activation, and proinflammatory cytokine production [32]. Additionally, autophagy plays an important role in kidney related inflammatory injury via NF-κB activation induced by lipopolysaccharide [33], and in an early host antifungal response by enhancing NF-κB activity through A20 sequestration [34]. These findings suggest that autophagy might participate in the regulation of inflammatory factors by activating the NF-κB pathway. Our study showed that altered *FHL2* expression did not affect changes in LC3B expression, but that *FHL2* expression was inhibited following treatment with the autophagy inhibitor CQ. This result suggested that autophagy is likely an upstream regulatory factor of the *FHL2*/NF-κB/IL-6 pathway. However, some TFs involved in orchestrating inflammatory responses have also been implicated in transcriptional regulation of core genes associated with autophagy. Prototypical examples of these TFs include NF-κB p65/RelA family members shown to upregulate Beclin-1 mRNA and protein levels in different cellular systems. Moreover, p65-mediated upregulation of Beclin-1, as well as IL-6 secretion, is coupled to increased autophagy [35,36]. Therefore, the existence of potential feedback regulation between autophagy and *FHL2*/NF-κB/IL6 status following PM2.5 exposure requires further investigation.

## 4. Materials and Methods

### 4.1. PM2.5 Collection and Preparation

Ambient PM2.5 was sampled at a flow rate of 77.59 L/min on a glass fiber filter (Whatman 41; Whatman, Maidstone, UK) by a mass flow meter (TSP/PM10/PM2.5-2; Beijing Geological Instrument-Dickel Co, Ltd, Beijing, China) at an altitude of 40 m between the 2nd and 3rd ring road of Beijing city, from October to December 2015. The filter was immersed in deionized water after sampling, from which the sample was eluted. The filter was cut into small pieces with an area of 1 cm^2^, immersed in sterilized water, and then sonicated three times to extract the water-soluble components using an ultrasonic cleaner (TP250B; Tianpeng Electronics, Zhongshan, China), followed by lyophilization and storage at −20°C.

### 4.2. Chemical Analysis of PM2.5

Inorganic components were detected by inductively coupled plasma atomic emission spectroscopy (ULTIMA; Horiba Jobin Yvon, Palaiseau, France).

### 4.3. MAEC Culture

MAECs were obtained from ATCC (Manassas, VA, USA) and were maintained in Dulbecco’s modified Eagle medium (DMEM) supplemented with 10% fetal bovine serum (FBS) at 37 °C in 5% CO_2_ atmosphere.

### 4.4. Enzyme-Linked Immunosorbent Assay (ELISA)

MAECs were seeded into 6-well plates and cultured to 70% to 80% confluence, followed by exposure to PM2.5 at different doses. The supernatants were collected after 24 h to analyze the release of IL-6 in response to PM2.5 treatment. IL-6 production in the supernatants was quantified using a mouse IL-6 high sensitivity ELISA kit (eBioscience; Thermo Fisher Scientific, Waltham, MA, USA).

### 4.5. PM2.5 Exposure and Cell Transfection

A certain quantity of PM2.5 was weighed and suspended in DMEM supplemented with 2% FBS, followed by homogenization for 30 min by an ultrasonicator. Cells were then incubated with different concentrations of PM2.5 for 24 h, after which total proteins were extracted using RIPA lysis buffer. The cDNA target sequence of small interfering RNAs (siRNAs) specific for *FHL2* was CTGTGACTTGTACGCTAAGAA. After siRNA transfection for 48 h, cells were stimulated with PM2.5 at a final concentration 100 μg/mL for 24 h, followed by protein extraction. Transfections were performed using Lipofectamine 2000 (Life Technologies, Rockville, MD, USA) according to manufacturer instructions.

### 4.6. Autophagy Assay

Cellular autophagy was monitored by Western blot analysis of specific key proteins (microtubule-associated protein 1A/1B light-chain 3B (LC3B) and Beclin-1), confocal microscopy (LSM510 META; Zeiss, Oberkochen, Germany), and flow cytometry (FACSCalibur; BD Biosciences, Franklin Lakes, NJ, USA). The presence of autophagosomes in PM2.5-induced MAECs was assessed using the Cyto-ID autophagy detection kit (175-0050; Enzo Life Sciences, Farmingdale, NY, USA), which was used to monitor specific autophagic fluorescence signals by confocal microscopy, or to quantitatively measure autophagic fluorescence intensity by flow cytometric analysis. For the immunoblot assay, induction of cellular autophagy in response to PM2.5 exposure was determined according to an increase in the endogenous LC3B-II:LC3B-I ratio and upregulation of Beclin-1 expression. Autophagic flux was assessed by comparing the extent of LC3B-II accumulation following PM2.5 treatment in the presence or absence of concomitant use of CQ, an inhibitor that disrupts lysosomal function.

### 4.7. Western Blot Assay

Total proteins were extracted using RIPA lysis buffer, and samples containing equal amounts of protein were subjected to sodium dodecyl sulfate polyacrylamide gel electrophoresis and Western blot. The following antibodies were used for Western blot analysis: IκBα (Cell Signaling Technology, Danvers, MA, USA), phosphorylated IκBα (Cell Signaling Technology), p65 (Cell Signaling Technology), phosphorylated p65 (Cell Signaling Technology), phosphorylated p38 (Cell Signaling Technology), p38 (Cell Signaling Technology), phosphorylated Akt (Cell Signaling Technology), Akt (Cell Signaling Technology), phosphorylated ERK1/2 (Cell Signaling Technology), ERK1/2 (Cell Signaling Technology), *FHL2* (Abcam, Cambridge, UK), LC3B (Cell Signaling Technology), Beclin-1 (Cell Signaling Technology), β-actin (CWBiotech, Shanghai, China), and glyceraldehyde 3-phosphate dehydrogenase (GAPDH; CWBiotech, Shanghai, China).

### 4.8. Statistical Analysis

All data were expressed as the mean ± standard deviation (SD), and analyzed using Student’s *t* test and one-way analysis of variance. Statistical analyses were performed using SPSS version 13.0 (SPSS, Inc., Chicago, IL, USA), and a *p* ≤ 0.05 was considered statistically significant.

## Figures and Tables

**Table 1:** Air-quality monitoring parameters.

Pollutant	Range	
	Low Concentration	High Concentration
PM2.5	32.3 μg/m^3^	296.3 μg/m^3^
Al	248.1 ng/m^3^	265.6 ng/m^3^
Se	3.8 ng/m^3^	13.8 ng/m^3^
Fe	324 ng/m^3^	704.4 ng/m^3^
Mn	17 ng/m^3^	55.1 ng/m^3^
Hg	0.7 ng/m^3^	1.2 ng/m^3^
As	3.7 ng/m^3^	12.5 ng/m^3^
Zn	101.1 ng/m^3^	318.4 ng/m^3^
Cu	12.4 ng/m^3^	34.2 ng/m^3^
Cd	0.7 ng/m^3^	3.6 ng/m^3^
Pb	39 ng/m^3^	157.4 ng/m^3^
Ni	1.0 ng/m^3^	3.1 ng/m^3^
Cr	1.8 ng/m^3^	4.9 ng/m^3^

## Abbreviations

*FHL2*	Four and a half LIM domains 2
NF-κB	Nuclear factor-κB
siRNA	Small interfering RNA
MAEC	Mouse aortic endothelial cells
PM2.5	Particulate matter with an aerodynamic diameter less than or equal to 2.5 μm
MCP-1	Monocyte chemoattractant protein 1
MIP-1α/β	Macrophage inflammatory protein 1α/β
IL-6	Interleukin-6
IL-1β	Interleukin-1β
sICAM-1	Soluble intercellular adhesion molecule 1
CVD	Cardiovascular diseases
PAH	Polycyclic aromatic hydrocarbon
FBS	Fetal bovine serum
LC3B	Microtubule-associated protein 1A/1B light-chain 3B
ERK1/2	Extracellular signal-regulated kinases 1/2
